# Orthophosphate binding at the dimer interface of *Corynebacterium callunae *starch phosphorylase: mutational analysis of its role for activity and stability of the enzyme

**DOI:** 10.1186/1471-2091-11-8

**Published:** 2010-01-29

**Authors:** Mario Mueller, Bernd Nidetzky

**Affiliations:** 1 Institute of Biotechnology and Biochemical Engineering, Graz University of Technology, Petersgasse 12, A-8010 Graz, Austria

## Abstract

**Background:**

Orthophosphate recognition at allosteric binding sites is a key feature for the regulation of enzyme activity in mammalian glycogen phosphorylases. Protein residues co-ordinating orthophosphate in three binding sites distributed across the dimer interface of a non-regulated bacterial starch phosphorylase (from *Corynebacterium callunae*) were individually replaced by Ala to interrogate their unknown function for activity and stability of this enzyme.

**Results:**

While the mutations affected neither content of pyridoxal 5'-phosphate cofactor nor specific activity in phosphorylase preparations as isolated, they disrupted (Thr^28^→Ala, Arg^141^→Ala) or decreased (Lys^31^→Ala, Ser^174^→Ala) the unusually strong protective effect of orthophosphate (10 or 100 mM) against inactivation at 45°C and subunit dissociation enforced by imidazole, as compared to wild-type enzyme. Loss of stability in the mutated phosphorylases appeared to be largely due to weakened affinity for orthophosphate binding. Binding of sulphate mimicking the crystallographically observed "non-covalent phosphorylation" of the phosphorylase at the dimer interface did not have an allosteric effect on the enzyme activity.

**Conclusions:**

The phosphate sites at the subunit-subunit interface of *C. callunae *starch phosphorylase appear to be cooperatively functional in conferring extra kinetic stability to the native dimer structure of the active enzyme. The molecular strategy exploited for quaternary structure stabilization is to our knowledge novel among dimeric proteins. It can be distinguished clearly from the co-solute effect of orthophosphate on protein thermostability resulting from (relatively weak) interactions of the ligand with protein surface residues.

## Background

α-(1,4)-D-Glucan phosphorylases (GlgP) promote degradation of glycogen, starch or maltodextrins by catalyzing glucosyl transfer from the non-reducing end of the glucosidic substrate to orthophosphate. They often serve a physiological function in fuelling the energy metabolism of the cell with α-D-glucose 1-phosphate (G1P) [1]. Although categorized as glycosyltransferases [2], GlgPs are special among enzymes of this class in that their activity is absolutely dependent on a pyridoxal 5'-phosphate (PLP) cofactor [3-5]. The PLP forms a Schiff-base linkage with ε-NH_2 _of an invariant Lys in the active site. The 5'-phosphate moiety is the cofactor group participating in catalysis [3,4,6]. All known GlgP enzymes are naturally active as dimers of two identical PLP-containing subunits [7-9]. Dimeric structure formation results in marked stabilization of the otherwise chemically labile protein-cofactor bond such that PLP is not detectably dissociable from native phosphorylase dimers [7,10,11]. GlgP enzymes in which activity is under control of covalent phosphorylation and/or allosteric effectors respond to regulatory signals through extensive rearrangements of their intersubunit contacts [5,12,13]. The dimer interface therefore is a key element of GlgP structure and function. While the overall pattern of subunit-subunit interactions is conserved in GlgPs, the molecular details vary among individual enzymes [5,7-9,13].

**Figure 1:**
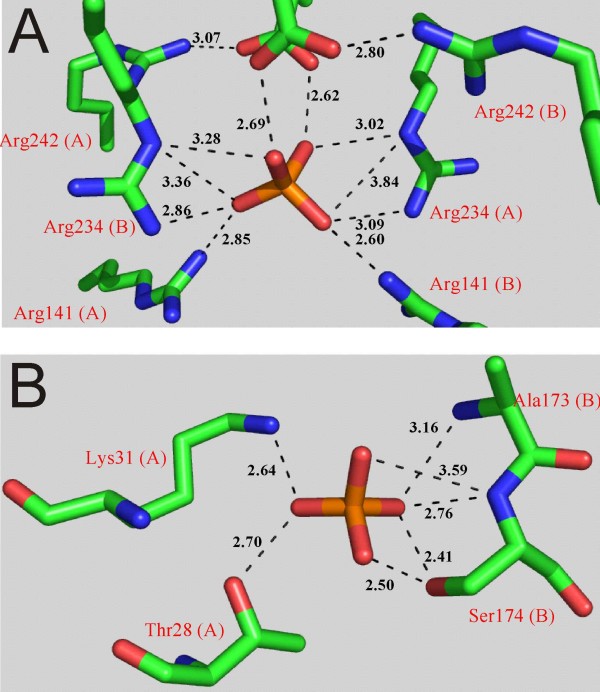
**Close-up structures of the orthophosphate binding sites at the dimer interface of *Cc*GlgP**. Panels A (Tower helix region; P-site) and B (CAP region) are drawn using the x-ray structure of *Cc*GlgP (PDB-accession code 2C4M). There are two identical CAP sites at the subunit-subunit interface, related by the internal symmetry of the enzyme dimer. H-bond distances are in Å, and coordinating residues contributed from subunit A or B are indicated in brackets.

*Corynebacterium callunae *starch phosphorylase (*Cc*GlgP) differs from related GlgP enzymes in that its dimer structure is highly susceptible to dissociative denaturation in the absence of external orthophosphate [14]. We interpreted evidence from detailed biochemical studies of the wild-type phosphorylase in terms of orthophosphate binding at a protein site (P-site) distinct from the catalytic site. Occupancy of the P-site was thought to result in substantially stabilized intersubunit contacts. Results of mutagenesis experiments located residues responsible for orthophosphate-dependent stability in the region of the so-called TOWER helix, a conserved secondary structural element of the dimer interface in GlgP enzymes [11,15,16]. The crystal structure of *Cc*GlgP at 1.9 Å resolution (PDB code: 2C4M) now reveals that not one but three orthophosphate sites are dispersed across the dimer contact region of the enzyme. The predicted P-site involving TOWER helix residues Arg^234 ^and Arg^242 ^is seen to bind orthophosphate in the structure. Analogous residues from neighbouring subunits in the *Cc*GlgP dimer form a symmetrical, highly positively charged interfacial binding site where Arg^141 ^and Arg^234 ^are co-ordinating and intersubunit contacts are established through a bound orthophosphate ion (Figure 1A). The two other, entirely novel binding sites for orthophosphate are located in the so-called CAP region of the dimer interface (Figure 1B). Dimerisation via the CAP helix (residues 17 - 28 in *Cc*GlgP) is a common structural feature among GlgP enzymes [5,7-9,13]. Although the CAP sites for orthophosphate binding in *Cc*GlgP show less positive charge density than the P-site, their occupancy with ligand would be expected to also result in substantially strengthened dimer contacts. Strikingly, orthophosphate binding at the CAP sites occurs near to where regulated GlgP enzymes become phosphorylated or bind the phosphate group of allosteric effectors like AMP or D-glucose 6-phosphate [5,9,12,13]. We thus speculated that orthophosphate binding at the CAP sites might be a novel mechanism by which *Cc*GlgP regulates or finely tunes its activity. However, the CAP sites of *Cc*GlgP appear to be structurally unrelated to known allosteric sites for orthophosphate in GlgP enzymes.

Extensive use of intersubunit binding of orthophosphate to stabilize the native dimer in *Cc*GlgP has not been described for other GlgP enzymes and appears to generally lack precedence in oligomeric proteins. We therefore used mutational analysis to determine the role of individual phosphate site residues for orthophosphate-dependent stability and activity in *Cc*GlgP.

## Methods

Unless mentioned otherwise, all materials used were described in previous papers [11,15-17].

### Site-directed mutagenesis, enzyme production and purification

The plasmid pQE 30-GlgP harbouring the gene encoding wild-type *Cc*GlgP fused to an N-terminal metal affinity peptide (RGSHHHHHHGSA) [15] was used as template for site-directed mutagenesis. Mutations were introduced by employing a modified two-stage PCR protocol [18] in which the following pairs of oligonucleotide primers (Invitrogen) were used with mismatched codons underlined. T28A: 5'-ACCTCGCTGCTGATCGCAAG-3' (forward primer), 5'-AGAACTTGCGATCAGCAGCGAG-3' (reverse primer); K31A: 5'-CTACTGATCGCGCGTTCTGGACTG-3' (forward primer), 5'-CAGTCCAGAACGCGCGATCAGTAG-3' (reverse primer); R141A: 5'-TGGTCTGCTCTACGCCTTCGGTC-3' (forward primer), 5'- AGACCGAAGGCGTA-GAGCAGACC-3' (reverse primer); S174A: 5'-TCGTGCAGCCGACCAGTTG-3' (forward primer), 5'-TGGTCGGCTGCACGACGAATAG-3' (reverse primer). Plasmid vectors harbouring sequence-proven inserts (VBC Genomics) were transformed into *E. coli *JM109, and recipient strains were grown for recombinant protein production as reported previously [11]. Protein purification followed a published protocol [19] except that no heat treatment was used. Purity of the obtained protein preparations was assessed by SDS PAGE. Isolated enzymes were stored at 4°C at a concentration of 4.0 - 12 mg/ml in 50 mM potassium phosphate buffer, pH 7.0.

### Biochemical characterization of mutated CcGlgP

Phosphorylase activity was measured with a continuous coupled enzyme assay described elsewhere [15]. The Bio-Rad dye binding assay referenced against BSA was used for determination of protein concentrations. The PLP content of isolated protein preparations was quantitated using a reported colorimetric method [20].

### Orthophosphate-dependent stability

Activity loss at elevated temperature. Protein solutions (22 - 53 μg/ml) were prepared in 50 mM triethanolamine buffer, pH 7.0 or 6.6, containing 24 mM KCl, 10.0 mM or 100 mM K_2_HPO_4_. Incubations were carried out in 1.5 ml tubes at 45°C. Samples (10 μl) were taken after 15 sec and then in regular intervals, depending on the stability of the enzyme used. They were diluted immediately into the continuous coupled assay of phosphorylase activity. Test for reversibility of inactivation involved cooling of the sample to room temperature followed by a 1 h-long incubation in the presence of 50 mM potassium phosphate, pH 7.0, and then activity measurement.

Inactivation by imidazole. A buffer (pH 7.0) containing 0.4 M imidazole and 0.1 M L-cysteine hydrochloride was used. K_2_HPO_4 _or (NH_4_)_2_SO_4 _was optionally added in a concentration of 5.0 mM. The protein was diluted to a final concentration of 22 - 53 μg/ml in the above-described buffer, and incubations were carried out at 30°C. Samples were taken at the times indicated and residual activity was measured using the continuous assay. Restoration of activity in partially imidazole-denatured preparations of the wild-type enzyme was examined in the presence of 200 mM K_2_HPO_4 _(pH 7.0) and 500 μM PLP.

### Gel filtration analysis

Size exclusion chromatography was performed using a BioLogic Duo-Flow System (model 2128; Bio-Rad, Hercules, U.S.A.) equipped with a HiLoad 16/60 Superdex 200 prep grade column (GE Healthcare). The column was equilibrated with 50 mM potassium phosphate buffer, pH 7.0, containing 0.15 M NaCl. It was operated with the same buffer using a flow rate of 1 ml/min. Gel Filtration Standard from Bio-Rad was employed for calibration. The applied sample (2 ml) typically contained = 0.8 mg of native or partially denatured protein.

### Effect of sulphate on α-glucan-synthesizing activity of wild-type and mutated forms of *Cc*GlgP

Initial rate measurements were performed in the direction of α-(1,4)-D-glucan synthesis at 30°C using a concentration of 2.5, 25 and 50 nM for the respective enzyme subunit. Reactions were carried out in 50 mM triethanolamine buffer, pH 7.0, with and without 5.0 - 40 mM (NH_4_)_2_SO_4 _present. Maltodextrin DE19 (AGENAMALT 20.235, Agrana, Austria) or soluble starch was used as acceptor substrate (20 g/l). The concentration of orthophosphate (P_i_) released from G1P (5.0, 20, and 50 mM) was measured in a minimum of three samples taken after 4 to 120 min using an assay described elsewhere [21]. The rate was calculated from the linear relationship of [P_i_] against time.

## Results and Discussion

### Selection of residues for site-directed substitution and properties of mutated enzymes

Considering results of previous studies delineating the disruptive effect of individual site-directed substitutions of Arg^234 ^and Arg^242 ^by Ala (R234A, R242A) on orthophosphate-dependent stability of *Cc*GlgP [15], we here selected Arg^141 ^from the P-site for mutational analysis. Figure 1A shows that Arg^242 ^has indirect interactions with orthophosphate via the co-ordinating Glu^235^. Because biochemical data for R242A clearly underscore the importance of the Glu^235^/Arg^242 ^couple for stabilization by orthophosphate [11,15], the Glu^235 ^was not further pursued here. Thr^28^, Lys^31^, and Ser^174 ^whose side chains co-ordinate orthophosphate at the CAP site (Figure 1B) were replaced by Ala. Purified preparations of the mutated enzymes migrated as single protein bands in SDS-PAGE, each showing the expected molecular mass of ~90 kDa (data not shown). Isolated phosphorylases contained ≥ 0.60 mol PLP per each mol of protein subunit. Their turnover numbers in the direction of phosphorolysis of maltodextrin (*k*_cat _≈ 40 - 47 s^-1^) were similar. Table 1 summarizes the data.

**Table 1:** Biochemical properties of wild-type and mutated forms of *Cc*GlgP.

	PLP	activity	
		
	[μM/μM protein]	[U/mg]	*k*_cat _[s^-1^]
wild-type	0.7	31	47

T28A	0.6	29	44
K31A	0.6	29	44
S174A	0.7	29	44

R141A	0.7	27	40

Activities were measured at 30°C at pH 7.0 in a continuous coupled enzyme assay in the presence of 50 mM P_i _and 24 g/l maltodextrin (Agenamalt 19DE). The *k*_cat _value was calculated from the specific activity (U/mg) assuming a molecular mass of 90.6 kDa for the enzyme subunit. PLP values have S.D. of < 20% (N = 3) while activity values have S.D. of < 10% (N = 4).

### Orthophosphate-dependent stability at elevated temperature

**Figure 2:**
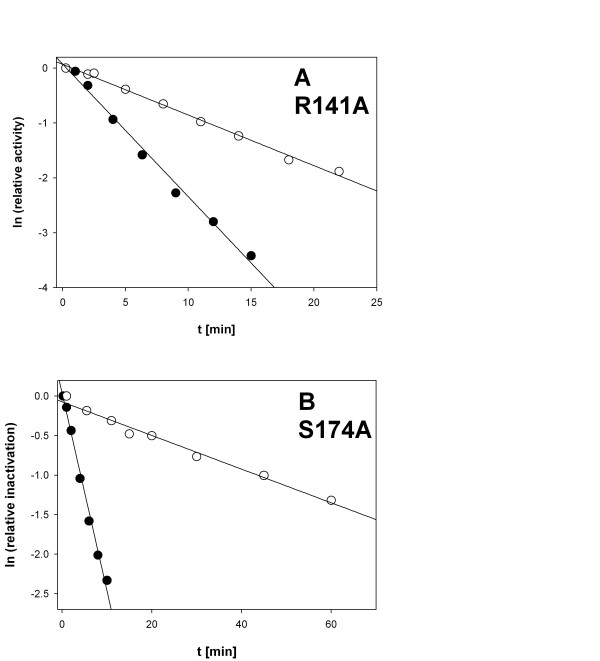
**Inactivation of R141A and S174A at 45°C**. A 50 mM triethanolamine buffer, pH 7.0, was used. The protein concentration was 35 μg/ml (A) and 20 μg/ml (B). Symbols show the experimental data and solid lines are the corresponding straight-line fits. Representative data are shown. Conditions: no P_i _added (full circles); 10 mM P_i _(open circles).

Incubation of purified phosphorylases at 45°C caused a time-dependent loss of enzyme activity that was kinetically first-order (Figure 2) and not detectably reversible under the conditions used. Previously reported data for wild-type enzyme and P-site mutants R234A and R242A have delineated a formal kinetic mechanism of denaturation at elevated temperature and indicated that loss of the native dimer structure due to subunit dissociation determines the observable rate of irreversible inactivation [14,15]. Note, however, that the phosphorylase monomer is not stable due to release of the PLP cofactor into solution, further unfolding and irreversible aggregation under conditions of temperature-induced denaturation [14]. Measurement of formation of phosphorylase monomer was therefore not considered in our experiments. It was also shown in prior studies of the wild-type enzyme and selected mutants thereof [14,16] that CD and Fourier-transform infrared spectroscopy can be used to portray the structural stabilization of *Cc*GlgP by orthophosphate. However, because evidence obtained through the spectroscopic techniques essentially confirmed the results of activity measurements [14-16], we decided to use enzyme activity as the most sensitive and also convenient reporter of phosphorylase denaturation by subunit dissociation.

Half-life times (*t*_1/2_) for wild-type and mutated phosphorylases were determined from linear fits of data recorded in the absence and presence of orthophosphate (Figure 2). They are summarized in Table 2. Under conditions in which orthophosphate was lacking, wild-type and mutated enzymes showed relatively similar stabilities (~7-fold difference overall). We note, however, that T28A was about 3 times less stable than the wild-type enzyme. The *t*_1/2 _of K31A was slightly enhanced compared to *t*_1/2 _of wild-type phosphorylase. Interestingly, therefore, the more than 100-fold enhancement of *t*_1/2 _that was caused by 10.0 mM P_i _in the wild-type enzyme was partly lost in K31A (only 30-fold stabilization), more so in S174A (only 12-fold stabilization), and essentially eliminated in T28A and R141A (only 3-fold stabilization).

**Table 2:** Comparison of half-life times (*t*_1/2_) of recombinant wild-type *Cc*GlgP and site-directed enzyme variants in thermal denaturation experiments and in the presence of 0.4 M imidazole at 30°C

t _(1/2) _[min]	inactivation at 45°C			inactivation by imidazole at 30°C	
	no P_i_	10.0 mM P_i_	100 mM P_i_	no P_i_	5.0 mM P_i_
wild-type	2.6	2.8 × 10^2^	3.5 × 10^3^	22	160
T28A	0.8	2.2	3.3 × 10^2^	1.9	5.6
K31A	5.3	1.7 × 10^2^	3.5 × 10^3^	10	25
S174A	2.8	33	9.9 × 10^2^	14	58
R141A	2.9	7.5	1.4 × 10^3^	6.4	27

The experiments were carried out in 50 mM triethanolamine buffer, pH 7.0. Values from linear regression analysis have S.D. of < 10%. Representative data were used for calculation of *t*_1/2_.

Decreased affinity for orthophosphate binding or intrinsically lowered stability of the protein-orthophosphate complex could explain the loss in orthophosphate-dependent stability of the *Cc*GlgP mutants. To distinguish between these possibilities, we determined *t*_1/2_for wild-type and mutated phosphorylases in the presence of 100 mM P_i _(Table 2). T28A and R141A were stabilized by a factor of 150 and 187, respectively, as compared to the corresponding *t*_1/2 _determined at 10.0 mM P_i_. Enhancement of *t*_1/2 _resulting from the increase in P_i _concentration was 21- and 30-fold in K31A and S174A, respectively, and can be compared to a 13-fold effect on *t*_1/2_for the wild-type phosphorylase. Because differences in stability among the individual phosphorylases seen at 10.0 mM P_i _were, to a very substantial extent, removed at 100 mM P_i_, we believe that it was mainly the P_i _binding affinity of the respective site (not the mechanism of stabilization) that was influenced by the chosen single point mutations. We did not test higher concentrations of orthophosphate than 100 mM because under these conditions, it is exceedingly difficult to distinguish the stabilization resulting from specific binding at a defined phosphate site from another stabilization due to non-specific protein-orthophosphate interactions [14]. Part of the enhancement of *t*_1/2 _for the wild-type phosphorylase upon increasing the orthophosphate concentration from 10.0 to 100 mM could already reflect non-specific stabilization.

The results in Table 2 show that structural modification of either phosphate site (e.g. R141A and T28A) can result in a nearly complete loss of orthophosphate-dependent stability at 10.0 mM P_i_. This finding suggests that the CAP-sites (where Thr^28 ^is located) and the P-site (where Arg^141 ^is located) do not function independently one from another, be it that orthophosphate binding at the two sites is truly cooperative or occupancy of *both *sites is a critical requirement for dimer stability. In the case that orthophosphate binding at each binding site made an independent contribution to the kinetic stability of *Cc*GlgP (measured as *t*_1/2 _at 45°C), one would expect that site-directed mutagenesis of one binding site causes only partial disruption of orthophosphate-dependent stability, which is contrary to observations for T28A and R141A.

### Orthophosphate-dependent stability in the presence of imidazole

**Figure 3:**
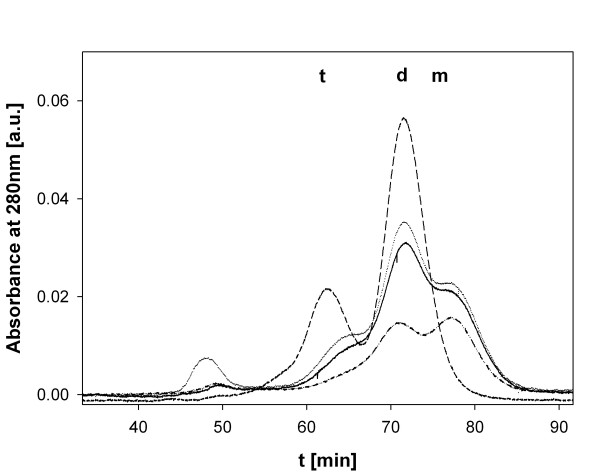
**Dissociation of enzyme subunits in R141A enforced by imidazole**. R141A as isolated is a mixture consisting of the native dimer and a small amount of a tetrameric form that is also active. The N-terminal His-tag causes the tetramerization [15]. The absorbance traces are in arbitrary units (a.u.). Elution profiles are shown for R141A prior to (dashed line) and after incubation in the presence of 0.4 M imidazole for 30 min (dotted line), 60 min (solid line) and 140 min (dashed-dotted line). The observed peaks correspond to tetrameric (**t**; 362 kDa), dimeric (**d**; 181 kDa) and monomeric (**m**; 90.6 kDa) forms of the protein. A high-molecular mass peak is also visible in some traces, presumably showing soluble aggregated protein. Loss of phosphorylase activity in the presence of imidazole is correlated with the extent to which monomer formation had occurred (data not shown). Note that all samples contained the same protein concentration (0.4 mg/ml) prior to the incubation with imidazole. The decrease in peak area for the eluted protein forms as the incubation time in the presence of imidazole increased probably reflects loss of protein due to aggregation. Insoluble aggregates are removed by centrifugation prior to gel filtration. The protein concentration of the sample applied to the Superdex column was not measured.

Assay conditions were used which in the wild-type phosphorylase lead to formation of a stable, monomeric apo-enzyme [11]. The presence of the His-tag does not alter the denaturation behaviour of the native *Cc*GlgP in the presence of imidazole. Loss of enzyme activity in the assay reflects dissociation of the protein subunits and is partly reversible upon addition of external PLP [11]. Results of time course experiments comparing wild-type enzyme and the various mutants revealed that the process of inactivation in each enzyme was kinetically first-order (data not shown), and Table 2 summarizes *t*_1/2 _values for denaturation by imidazole in the absence and presence of 5.0 mM orthophosphate. The stability of the wild-type enzyme was slightly lower than reported previously which is explicable on account of a 6-fold lower protein concentration used in the experiments described herein, as compared to literature [11]. It was confirmed that addition of PLP restored activity in partly inactivated enzyme preparations, and time-dependent conversion of the native R141A dimer into an inactive monomer was demonstrated by using size exclusion chromatography (Figure 3).

With the exception that all mutated phosphorylases, however especially T28A, were less stable than the wild-type enzyme under conditions where 5.0 mM orthophosphate was lacking, data confirm the overall trend seen in inactivation experiments at 45°C that the mutations decreased the stabilizing effect of orthophosphate in the wild-type enzyme. Generally, orthophosphate was much less stabilizing to denaturation by imidazole than denaturation by heat (45°C). However, we must consider that, while both methods of denaturation promote dissociation of subunits in the phosphorylase dimer [11,15], their effects on the protein structure are probably not identical. The disruptive effect of the mutations on orthophosphate-dependent stability was smaller when using imidazole as compared to 45°C as trigger of denaturation.

### Allosteric effect of orthophosphate binding on enzyme activity?

Initial rates of α-(1,4)-glucan synthesis (*V*_s_) catalyzed by native *Cc*GlgP were recorded in the absence and presence of (NH_4_)_2_SO_4_. Previous work has shown that sulphate is similarly efficient as orthophosphate in stabilizing the dimer structure of *Cc*GlgP [11,14], validating the use of sulphate as an orthophosphate surrogate in kinetic experiments. Note the added orthophosphate would have interfered with the assay applied for determination of *V*_s_.

**Figure 4:**
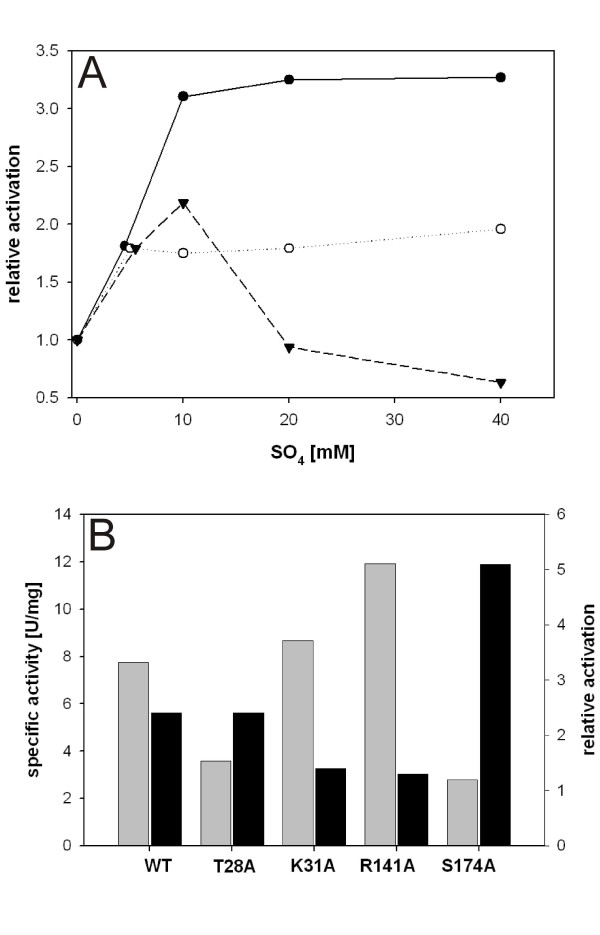
**Enhancement by sulphate of enzyme activity in the synthesis direction for wild-type and mutated forms of *Cc*GlgP**. A: Effect of sulphate on *V*_s _for the wild-type phosphorylase. (full circles), 25 nM protein, 50 mM G1P; (full triangles) 25 nM protein, 5.0 mM G1P; (open circles), 50 nM protein, 50 mM G1P. Starch (20 g/l) was the acceptor substrate. B: Specific activities (grey bars; left y-axis) for wild-type and mutated phosphorylases determined at a protein concentration of 2.5 nM in the absence of (NH_4_)_2_SO_4 _using 50 mM G1P and 20 g/l maltodextrin as substrates. Relative enhancement of the respective activity by 10 mM (NH_4_)_2_SO_4 _is shown with black bars (right y-axis). All results are representative data.

We observed a significant enhancement of *V*_s _in dependence of the sulphate concentration. Figure 4A shows the degree of "activation" of the enzyme relative to the control lacking sulphate. Using a saturating concentration of G1P (50 mM) in the assay, stimulation of *V*_s _was a maximum (~3-fold) at around 10 mM sulphate and remained constant as the oxyanion concentration was further increased. Inhibition by high concentrations of sulphate under conditions where a non-saturating level of αG1P (5 mM; *K*_m _= 1.04 mM [22]) was employed, is plausibly explained by competition between substrate and oxyanion for binding to the active site. The extent to which sulphate stimulated *V*_s _decreased as the protein concentration in the assay was increased, suggesting that the observed "activation" by sulphate was apparent and likely reflected stabilization of the functional *Cc*GlgP dimer at the low protein concentrations used in the assay. The dissociative mechanism of denaturation of *Cc*GlgP which applies to a wide range of conditions including room temperature [14,16] implies that the enzyme is more stable at high protein concentrations.

Figure 4B shows a comparison of the effect of 10 mM sulphate on *V*_s _for wild-type and mutated forms of *Cc*GlgP measured at a protein concentration of 2.5 nM. The concentration of αG1P was 50 mM. The 2.4-fold apparent activation of the wild-type enzyme under these conditions was retained in T28A whereas it was almost completely lost in K31A and R141A. The strong (~5-fold) enhancement of activity of S174A at the low protein concentration was attenuated to a 1.7-fold "activation" at a higher protein concentration of 50 nM. Addition of sulphate partly eliminated differences in specific activity between the individual enzymes observed under conditions where the oxyanion was lacking. However, the concentration of sulphate required to raise the specific activity of T28A to the level of the wild-type enzyme was higher than 10 mM, and full complementation of the mutated phosphorylase was obtained at 40 mM oxyanion. These results agree with the notion (Table 2) that orthophosphate/sulphate binding affinity at the CAP site was strongly decreased as result of individual substitutions of Thr^28 ^by Ala. They also concur with the proposed mechanism of action of sulphate where stimulation of activity is apparent and derives from a stabilized dimer structure. A possible allosteric effect of interfacial orthophosphate/sulphate binding on the enzyme activity is therefore not supported.

## Conclusions

The CAP- and P-sites for orthophosphate binding at the subunit-subunit interface of *Cc*GlgP appear to be cooperatively functional in conferring extra kinetic stability to the native dimer structure of the active enzyme. The molecular strategy exploited for quaternary structure stabilization is to our knowledge novel among dimeric proteins. It can be distinguished clearly from the co-solute effect of orthophosphate on protein thermostability resulting from (relatively weak) interactions of the ligand with protein surface residues, often lysines [23,24]. However, *Treponema denticola *cystalisin is an interesting example of a PLP-containing enzyme that utilizes hydrogen bonding between the 5'-phosphate of the cofactor and a tyrosine from the respective other subunit to stabilize the functional protein homodimer [25]. We have shown here that Thr^28 ^at the CAP site of *Cc*GlgP is of key importance for orthophosphate-dependent stability of the enzyme. Therefore, although an allosteric effect of oxyanion binding on enzyme activity was not clearly supported by the data, it was nevertheless interesting that an unregulated phosphorylase has accommodated a functional phosphate site in a protein region where the principle of phosphate group recognition was exploited by nature to evolve the regulatory sites in today's eukaryotic α-(1,4)-D-glucan phosphorylases.

## Authors' contributions

M. M. carried out all experiments, analyzed data and drafted the paper. B. N. designed research, co-analyzed and interpreted data, and wrote the paper. Both authors have read and approved the manuscript.
